# Analysis of influencing factors of phenanthrene adsorption by different soils in Guanzhong basin based on response surface method

**DOI:** 10.1038/s41598-022-25293-0

**Published:** 2022-12-03

**Authors:** Hua Tian, Qing Zhang, Xue Tian, Zu-feng Xie, Fang Pu, Qian-ji Wang

**Affiliations:** 1 College of Geology and Environment, Xi’an University of Science and Technology, Xi’an, 710054 China; 2 Shaanxi Provincial Key Laboratory of Green Coal Development and Geological Guarantee, Xi’an, 710054 China; 3 Zhongsheng Environmental Technology Development Co, Ltd, Xi’an, 710054 China

**Keywords:** Ecology, Ecology, Environmental sciences

## Abstract

Adsorption desorption is an important behavior affecting the migration of phenanthrene in soil. In this study, three typical soils of loess, silts and silty sand in Guanzhong Basin, Shaanxi Province, China were used as adsorbents. Batch equilibrium experiments were carried out to study the adsorption desorption kinetics and isotherm of phenanthrene in different soils. Response surface method (RSM) was used to study the effects of temperature, pH, phenanthrene concentration and organic matter content on soil adsorption of phenanthrene. The results showed that after adsorption, the outline of soil particles became more blurred and the degree of cementation increased. The kinetic adsorption of phenanthrene by soil conforms to the quasi second-order kinetic model, and the adsorption desorption isotherm is nonlinear and conforms to the Freundlich model. Due to the difference of soil properties, the adsorption amount of phenanthrene by soil is loess > silty sand > silts. The thermodynamic results show that the adsorption of phenanthrene by soil is spontaneous and endothermic, and the desorption is spontaneous and exothermic. Through RSM, the interaction between phenanthrene concentration and soil organic matter in Loess and silts is significant, and the interaction between temperature and soil organic matter in silty sand is significant. Among the four factors affecting the adsorption rate of loess, silts and silty sand, soil organic matter is the most significant. The theoretical optimum adsorption rates of loess, silts and silty sand are 98.89%, 96.59% and 93.37% respectively.

## Introduction

Polycyclic aromatic hydrocarbons (PAHs) are a kind of toxic and harmful organic pollutants widely distributed in the natural environment where the soil bears more than 90% PAHs in the environment^1–5^. A series of transport and transformation behaviors will occur when PAHs enters the soil, including adsorption, desorption, biodegradation, photodegradation et al. Adsorption and desorption play an important role in influencing PAHs in soil environments^6^. PAHs entering the soil environment can be easily absorbed by soils. At the same time the absorbed PAHs can also be desorbed with the solvent. Absorbed PAHs in soils will enter into the food chain through the plants metabolism, posing a serious threat to the ecological environment and human health^7,8^. Understanding the transport and fate of PAHs in soil environment is paramount to determinations of the PAHs risks to human health and the environment. It is also to help providing strategies for soil contamination.

The adsorption and desorption mechanisms of PAHs in soils were mainly focused on the kinetics and isotherms theory^9^. The adsorption and desorption kinetics of PAHs was considered to be completed in two processes. One was the rapid adsorption process, which took several minutes to several hours to reach equilibrium^10,13^. The other was the slow adsorption process, which took months to several years^14^. Some research showed that adsorption kinetics of phenanthrene on soils conformed to the first-order kinetic model. Others are more consistent with the pseudo-second-order model^15^. In addition, the adsorption isotherm of PAHs on soils could be described quantitatively by fitting experimental data with different adsorption isotherms. The most commonly used isotherms were Freundlich^16,17^, Langmuir^18^, Temkin^19^ and Brunauer–Emmett–Teller (BET) models^20^.

The adsorption and desorption of PAHs in soils can be influenced by different factors such as PAHs properties, soil type, mineral composition, pH, soil porous structure, organic matter et al. The adsorption of PAHs on particulate was affected by the molecular weight and hydrophobicity of PAHs. PAHs with a greater octanol–water partition coefficient (Kow) tended to be highly affected by partitioning into organic matter, resulting in more difficult desorption from soil^9^. It is recommended that adsorption and desorption behavior of PAHs in soils were mainly based on mineral component and organic matter^21,22^. The desorption of phenanthrene decreased with the organic content increasing^23^. This was because organic matter in soil enhanced the sorption and affinity of PAHs due to partitioning into the soil organic phase rather than clay minerals surfaces^24^. Soil organic matter could be divided into dissolve phase and pore filling phase. The former was linear distribution model and the latter followed the adsorption isotherm model. Some studies showed that pH affected the adsorption of PAHs on soils and the adsorption capacity would be decreased with the increased pH^25^. In addition, temperature was another important factor in the adsorption and desorption behavior of PAHs. Zhu studied the adsorption characteristics of naphthalene and phenanthrene in soil at three different temperatures (25, 35, 45 °C). It was found that the adsorption rate of naphthalene and phenanthrene were negatively correlated with temperature. Ren^26^ suggested that temperature would affect the solubility of PAHs in water, thereby affect the adsorption strength of PAHs. Shi^27^ showed that the adsorption of naphthalene in soil was an endothermic reaction, which was driven by entropy increased. But in most cases, the adsorption of the PAHs was an exothermic reaction.

Guanzhong basin is located in Shaanxi province, China. It is the center of Shaanxi province and the main industrial and agricultural area. With the economic development and population growth, the municipal wastewater and industrial waste discharge were greatly increased, leading to the organic pollution risk of soil. However, little is known about the mechanisms in PAHs adsorption and desorption on soils in Guanzhong basin. In addition, the soils in Guanzhong basin have different physical and chemical properties^28^. The characteristics of soils could influence the adsorption and desorption behaviors of PAHs. The adsorption and desorption capacity of the soil can directly affect the downward migration of pollutants from the surface layer. If the pollutants are retained in the soil, thereby affecting the mobility of the pollutants, the process of continuing the infiltration of the pollutants is blocked, and it is not easy to infiltrate. It can reduce the pollution of the soil and groundwater. The effect of soil on pollutants is different due to different soil adsorption capacity. However, comparative studies on the adsorption and fixation ability of different soils in the same area are rare, and the main focus is on the clay layer and other rocks. Comparative studies of sexual block strength are relatively rare. In this paper, three typical soils in Guanzhong basin, named loess, silts and silty sand, were chosen, while the adsorption and desorption characteristics of phenanthrene on these soils were studied, including kinetics, isotherm and thermodynamics of adsorption and desorption. The kinetics and equilibrium adsorption model were used to describe the adsorption and desorption characteristics. In addition, the BBD experimental design response surface method was used to study the influence of phenanthrene concentration, soil organic matter content, temperature, pH and their interaction on soil adsorption of phenanthrene, and to determine the optimal adsorption rate and the influencing factors under this condition. To study the environmental behavior of PAHs in soil and provide the basis for pollution control, and analyze their adsorption mechanism through Fourier transform infrared spectroscopy and scanning electron microscopy.

## Materials and method

### Instruments and reagents

Experimental reagents: phenanthrene (analytical pure, purity > 97%), purchased from Aladdin Industrial Company, methanol (chromatographically pure) from TEDA, calcium chloride, hydrochloric acid, sodium hydroxide and hydrogen peroxide were all analytically pure.

Experimental equipment: constant temperature water bath oscillator SHA-B, desktop centrifuge TGL-15B, high performance liquid chromatograph (Agilent 1260), constant temperature magnetic stirrer DF-101S, electronic balance LE204EI02, box-type resistance furnace SX2-4-10A.

### Soil characterization

The soil used in the experiment was selected from a vertical section of the Weihe River, and three different types of soils, namely loess plateau loess, first-order terrace silty sand and floodplain silts, were collected as the research object. The three typical soils were collected at a depth of 0–20 cm, and the sundries were removed. After air-drying and grinding, they were sieved with a 60-mesh sieve. The soil samples after sieving were stored in a sealed sample bag for future use. The basic properties of the soils are summarized in Table 1.

**Table 1:** Sampling stations of soil and their characteristics.

Property		Soil sample		
		Loess	Silts	Silty sandy
Station		108°E 34°N	107°E 34°N	107°E 34°N
Sand (%)		5.11	43.7	70.17
Silt (%)		78.77	53.11	18.11
Clay (%)		16.12	3.19	11.72
Density (g/cm^3^)		1.65	1.52	1.61
pH (water)		8.88	8.64	8.46
Water content (%)		0.24	0.14	0.19
Medium particle-D50 (μm)		14.81	32.215	45.52
Specific surface area (m^2^/g)		1.194	1.172	0.833
Soil organic matter (%)		1.2734	0.9411	0.1327
Phenanthrene content (mg/kg)		1.03	0.445	1.103
Soil constitutions (%)	Quartz	74.61	73.38	58.92
	Feldspar	25.2		
	Anorthose		26.2	13.92
	Mica			16.79
Chemical components	CaCO_3_	13.28	46.8	12.71
	SiO_2_	64.48	50.94	64.12
	Al_2_O_3_	3.81	1.21	5.41

In order to make the data clear, the coded values are used for organic matter.

Preparation of soil samples with different organic matter content: Weigh 10.00 g of each of the three soil samples into a conical flask, and burn them at 0 °C, 200 °C and 400 °C respectively. The organic matter content was determined according to GB/T642-1999 using potassium dichromate method. The specific operations are as follows:

Weigh 3 g soil samples respectively and put them into a dry conical flask, slowly drop 10 mL of potassium dichrochrome standard solution and shake well, add 1 mL of concentrated sulfuric acid, and put a small funnel into the test tube mouth to collect condensed liquid. Put the conical flask into the oven at about 185 °C, control the temperature of 170 ~ 180 °C to boil for five minutes, and take out a little cooler. Rinse the funnel and the inner wall of the conical flask with pure water, control the pure water within 10 mL, and then add 3 drops of Aphaline indicator. The ferrous sulfate standard solution was titrated until the solution changed from yellow to brownish red after green as the end point. The amount of ferrous sulfate standard solution was recorded and estimated to 0.05 mL. Then, the soil sample burned at 500 °C for 5 h was used to replace the sample and the above steps were repeated for blank test.

The formula for calculating organic matter is as follows:

1 $$O_{m} = \frac{{c\left( {Fe^{2 + } } \right)V\left( {Fe^{2 + } } \right) - V\left( {Fe^{2 + } } \right) \times 0.003 \times 1.724 \times \left( {1 + 0.01w} \right) \times 100}}{m},$$

where, *O*_*m*_ for organic matter content (%); *c(Fe*^*2*+^*)* for ferrous sulfate standard solution concentration (mol/L); *V(Fe*^*2*+^*)* is a blank titration ferrous sulfate dosage (mL); *V(Fe*^*2*+^*)* as the sample (ferrous sulfate dosage (mL); 0.003 is the molar mass kg/mol of 1/4 ferrous sulfate standard solution concentration; 1.724 is the factor of converting organic carbon to organic matter. The organic matter content under the conditions is shown in Table 2.

**Table 2:** Values of experimental factors for different types of soil adsorption and desorption systems.

The value of each level code	Phenanthrene Concentration/(mg·L^−1^) (A)	pH (B)	Temperature/℃ (C)	Loess organic matter (D1)	Silts organic matter (D2)	Silty sand organic matter (D3)
− 1	10	2	20	0.0064	0.0047	0.0006
0	20	6	30	0.6399	0.4729	0.0667
1	30	10	40	1.2734	0.9411	0.1327

### Adsorption and desorption experiments

Adsorption experiment was carried out using a batch procedure. An appropriate soil was transferred to centrifuge tube. 10 mg/L of Phenanthrene electrolyte (in background solution of 0.01 mol/L CaCl_2_ to maintain a constant ionic strength) was added to the tube. All tubes were immediately sealed and then mechanically shaken for 24 h in a thermostatic oscillation incubator (200 r/min) at 25 °C. In thermodynamic experiments, the temperatures were adjusted to 15 and 35 °C. Samples were taken at different times and then the suspensions were centrifuged at 8000 r/min for 3 min. A 2 mL supernatant was filtered through the membrane with a pore size of 0.22 μm and was then analyzed by HPLC. Each processing was set for three repetitions. A blank (no soil) was prepared for each time point. Phenanthrene loss during adsorption and filtration was negligible. To determine the contact time required to achieve sorption equilibrium, the suspensions were sampled at 0, 5, 10, 60, 120, 300, 480 and 1080 min^29,30^. The amount of Phenanthrene adsorbed was calculated by Eq. (2):

2 $$q_{e} { = }\frac{{\left( {C_{0} - C_{e} } \right)V}}{m},$$

where, *q*_*e*_ (mg/g) is the amount of phenanthrene absorbed; *C*_*0*_ (mg/L) and *C*_*e*_ (mg/L) are the initial and equilibrium aqueous concentrations, respectively; *V*(mL) is the solution volume; and *m*(g) is the mass of soil in the centrifuge tubes.

After adsorption experiments, desorption experiments were conducted by replacing supernatant with phenanthrene-free background solution after drying. The tubes were agitated (200 r/min) for 24 h and then centrifuged at 8000 r/min for 3 min. The supernatants were filtered through 0.22 μm syringe filters and analyzed by HPLC^31^.

Phenanthrene concentrations in the liquid phase were measured using a HPLC (Agilent 1260) equipped with a C18 analytical column (150 × 4.6 mm), and detected by UV spectrophotometry. The operating conditions were: mobile phase 90/10(V/V) of methanol/water, flow rate 1 mL/min, column temperature 35 °C, and detector wavelength 254 nm. The retention time for Phenanthrene was 8.1 min.

### Response surface methodology to optimize experimental design

In order to better use the response surface method to optimize the experimental conditions and study the adsorption behavior of different soil samples to phenanthrene, 4 variables of phenanthrene concentration (A), pH (B), temperature (C) and soil organic matter content (D) were selected in this experiment, respectively, single factor experiments were carried out to obtain the subsequent response surface adsorption equilibrium time and the parameter range of the experimental design. According to the previous experimental results, the adsorption equilibrium state can be reached after 18 h of soil adsorption of phenanthrene. Therefore, 18 h was selected as the adsorption equilibrium time in the subsequent experiments. Taking soil organic matter content of 0 (coded value), initial phenanthrene concentration of 20 mg/L, temperature of 30 °C and pH = 6 as the center points of the response surface experimental design. At the same time, according to the Box-Behnken (BBD) experimental principle, the 4-factor 3-level response surface method was used to explore the main effect and interaction of the above-mentioned 4 factors on soil adsorption of phenanthrene. The coding value of the experimental design is coded by Eq. (3):

3 $$x_{i} = \frac{{X_{I} - X_{0} }}{{\Delta X_{I} }},$$

where *x*_*i*_ is the coded value of the independent variable, *X*_*I*_ is the actual value of the independent variable, and *X*_*0*_ is the actual value of the central point of the independent variable, *ΔX*_*I*_ is the change value.

Through the data of various mathematical models (linear, two factor interaction, quadratic and cubic) and their variance analysis, it is shown that this method is most suitable for fitting with a quadratic polynomial model. The phenanthrene adsorption rate is selected as the response value, and its predicted value and independent variable are expressed by Eq. (4):

4 $$R = \beta_{0} + \sum\limits_{i = 1}^{n} {\beta_{i} x_{i} + \sum\limits_{i = 1}^{n - 1} {\beta_{ii} x_{i}^{2} + \sum\limits_{i = 1}^{n - 1} {\beta_{ij} x_{i} } } } x_{j} + \varepsilon ,$$

where *R* is the predicted response value of phenanthrene adsorption rate; *β*_*0*_ is the intercept coefficient; *β*_*i*_ is a linear parameter; *β*_*ii*_ and *β*_*ij*_ is interaction and second-order coefficient respectively; *x*_*i*_ and *x*_*j*_ are coded independent variables.

The coded and experimental values of the experimental design are shown in Table 2.In order to make the data clear, the coded values are used for organic matter.

### Data analysis

Pseudo-second-order kinetics (Eq. (5)) and the Elovich model (Eq. (6)) were used to fit (stimulate) the adsorption kinetics. The equations can be expressed as:

5 $$\frac{t}{{q_{t} }} = \frac{1}{{K_{2} q_{e}^{2} }} + \left( {\frac{1}{{q_{e} }} \times t} \right),$$

6 $$q_{t} = a + bt,$$

where, *q*_*t*_ (mg/g) is the amount of phenanthrene adsorbed at time *t*(min) and under equilibrium conditions, respectively; and *k*_*2*_ (g/(mg⋅min)) is the pseudo-second-order model rate constant for adsorption.

The Freundlich and Langmuir models were used to fit the adsorption and desorption isotherms. The two models were described dynamic equilibrium of adsorption, as the adsorption reaction proceeds. The adsorption site on the adsorbent surface was occupied constantly, making the adsorption rate gradually slow down. As the desorption rate was gradually accelerated in the process of adsorption, eventually the two rates were equal and the adsorption reaches the dynamic equilibrium. The Langmuir equation (Eq. (7)) is:

7 $$\frac{1}{{q_{e} }} = \frac{1}{q} + \left( {\frac{1}{{k_{L} q}}} \right)\left( {\frac{1}{{c_{e} }}} \right),$$

where, *q*(mg/g) is the amount of phenanthrene adsorbed by a mass of soil; and *K*_*L*_(L/mg) represents the Langmuir constant related to the bonding force of sorption.

Freundlich is an empirical equation, which is widely used in cases where adsorbent surface is not uniform and interaction between adsorbents exists. The Freundlich equation (Eq. (8)) is:

8 $$\log q_{e} = \log k_{F} + \left( \frac{1}{n} \right)\log c_{e} ,$$

where, *K*_*F*_((mg/g)(mg/L) − n) is the adsorption equilibrium constant representing the adsorption capacity; $n$ is a constant indicative of adsorption intensity.

The experiments were carried out at 293 K, 303 K, and 313 K. The thermodynamic parameters for the adsorption processes were obtained by the following equations (Eqs. (9), (10), (11)) ^32^:

9 $$\Delta G = - RT\ln \left( {\rho_{W} K_{F} } \right),$$

10 $$\Delta G^{0} = \Delta H^{0} - T\Delta S^{0} ,$$

11 $$\ln \left( {\rho_{W} K_{F} } \right)K_{F} = \frac{{\Delta H^{0} }}{RT} - \frac{{\Delta S^{0} }}{R},$$

where, *T*(K)is the absolute temperature; $R$ is the gas constant (8.314 J/(mol⋅K)); *ΔS*^*0*^ (kJ/(mol⋅K)) is the standard entropy change; *ΔG*^*0*^ (kJ/mol) is the standard Gibbs free energy;and *ΔH*^*0*^ (kJ/mol) is the standard enthalpy change; *ρ*_*w*_ is the water density (mg⋅L^−1^). *ΔH*^0^ and *ΔS*^*0*^ were obtained from the slopes and intercepts of a linear regression of lnK vs. $1/T$.

## Results and discussion

### Surface morphology analysis

**Figure 1:**
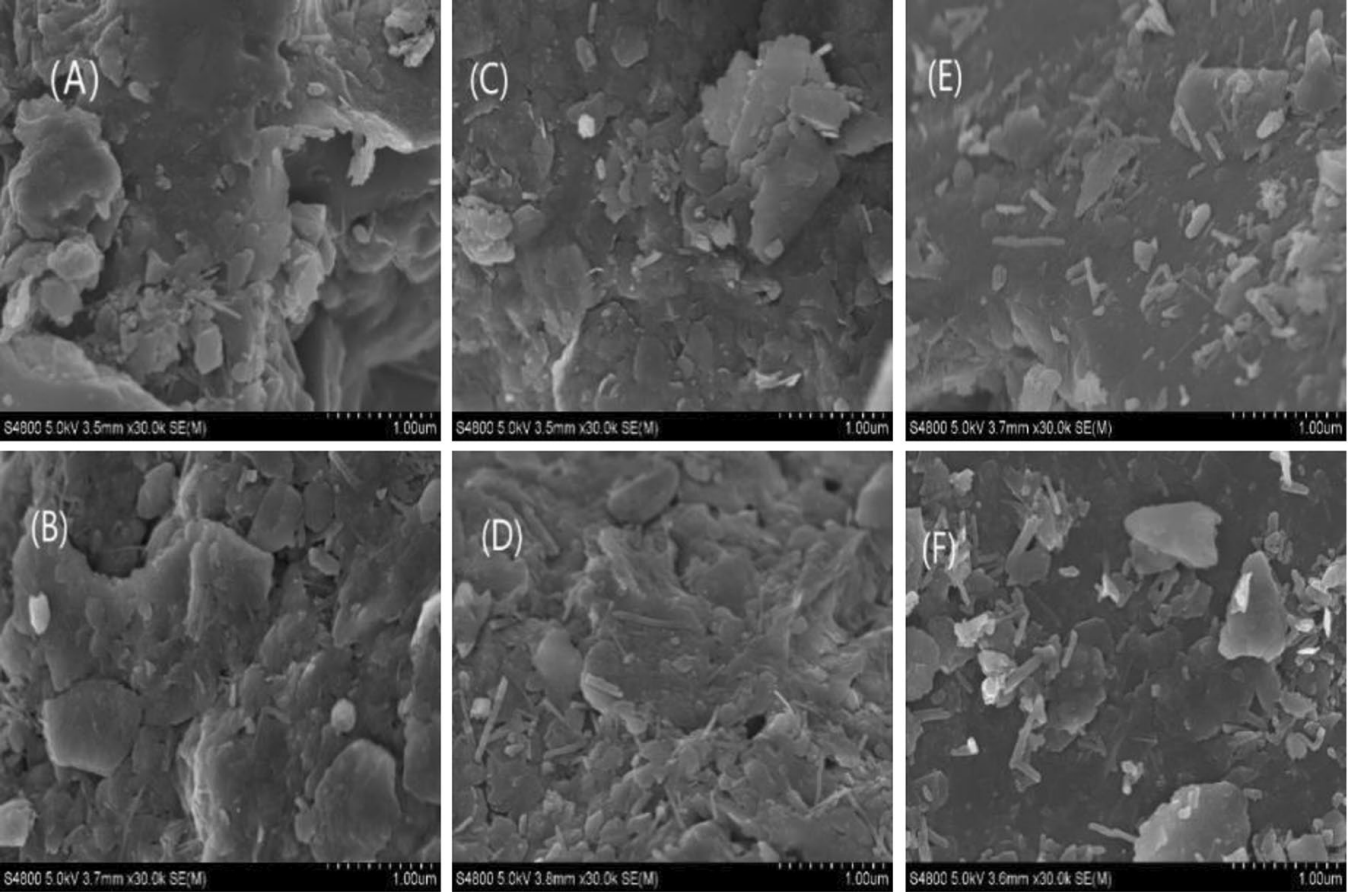
SEM micrographs of the three soil samples. (**A**) Silty sand; (**B**) Adsorbing 5 h of Silty sand; (**C**) Silts; (**D**) Adsorbing 5 h of Silts; (**E**) Loess; (**F**) Adsorbing 5 h of Loess.

SEM images were shown in Fig. 1. It showed that the contour of three soils were fairly clear before adsorption. But it became fuzzier and the degree of cementation was increased when phenanthrene was adsorbed on the soils. According to the surface morphology, the silty sand (A) had furrows on the surface before adsorption compared with the fairly smooth without any furrows after adsorption (B). The silts (C) were flaky and the lamellar accumulation decreased (D). The loess (E) had a smooth surface with some flaky and rod like structure, after adsorption (F), the surface of loess increased in clay-like structure.

### Adsorption and desorption experiments

#### Adsorption and desorption kinetics

**Figure 2:**
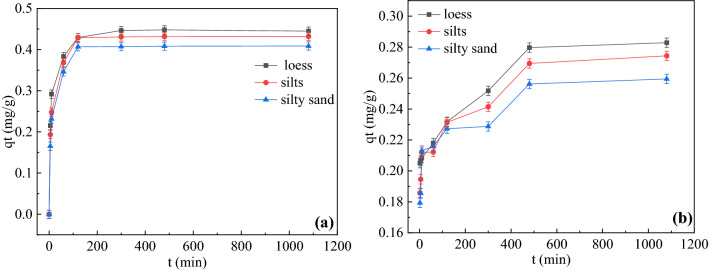
(**a**)Adsorption equilibration curves of phenanthrene sorption in soils. (**b**) Desorption equilibration curves of phenanthrene sorption in soils.

Adsorption kinetics is one of the most important characteristics governing solute uptake rate and represents adsorption efficiency^33^. The sorption and desorption kinetics of phenanthrene in three soils were shown in Fig. 2. The results showed that the adsorption processes among all soils were similar. The kinetics of phenanthrene in soils was completed in two steps: a “fast” adsorption and a “slow” adsorption. The adsorption amount increased during 0-18h. It was a rapid reaction from 0 to 200 minutes. From 200 to 600 minutes, the adsorption amount increased slightly into balance. This phenomenon was due to the adsorption of phenanthrene occurred on the surface of soil organic matter. With the increase of time, soil surface adsorption sites were gradually saturated, causing the decrease of adsorption rate until reaching the equilibrium. Phenanthrene was a hydrophobic substance. It was easy to reach the soil surface and adhere to the grain surface. The results were consistent with the study of had also found that the balance time was approximately 18h and the adsorption amount increased with the adsorption reaction time^34^. Under the same conditions, loess had the highest adsorption capacity, which was mainly due to the highest organic content 18. The maximum phenanthrene sorption capacities ranked as follows: loess > silty sand > silts. As shown in Fig. 2, phenanthrene desorption in soils was relatively quick and its desorption equilibrium time was 3h. To reach an adequate desorption balance while remaining consistent with the adsorption reaction time, the balance time of the adsorption–desorption experiment was set at 18h. Generally, PAHs below 4 cycles could reach the adsorption equilibrium for about 16~24h.

Pseudo-second-order and Elovich models were used to study the phenanthrene adsorption mechanism (Table 3). Phenanthrene sorption kinetics were satisfactorily described by a pseudo-second-order model with coefficients of determination (R^2^) ranging from 0.99875 to 0.99847, compared with R^2^ values of 0.26508–0.73901 for the Elovich model. This well-fitting pseudo-second-order model indicated that the rate-limiting step was chemical adsorption, including electronic forces through sharing or exchange of electrons^35,36^. Moreover, it suggested that sorption was governed by the availability of sorption sites on the soil surfaces instead of by the phenanthrene concentration in solution.

**Table 3:** Constants and coeffients of determination of Pseudo-second-order kinetics and Elovich models of sorption.

Sample	Study	Pseudo-second-order model			Elovich equation		
		*q*_*e*_(mg/g)	*K*_*2*_(g/(mg.min))	R^2^	a	b	R^2^
Loess	Adsorption	0.28574	0.2151	0.99847	7.41E–5	0.21786	0.73901
	Desorption	0.43192	0.1570	1	4E–06	0.4286	0.73435
Silts	Adsorption	0.2610	0.2913	0.99843	5.03E–5	0.21377	0.72497
	Desorption	0.4088	0.14808	1	4E–06	0.405	0.4924
Silty sand	Adsorption	0.2749	0.2128	0.99785	6.63E–5	0.21235	0.6729
	Desorption	0.4343	4.1928	1	2E–06	0.433	0.2658

#### Adsorption and desorption isotherms

**Figure 3:**
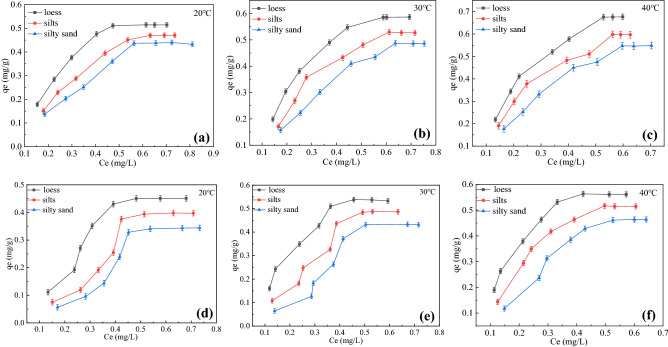
(**a**)20 °C adsorption isotherms for phenanthrene in soils. (**b**)30 °C adsorption isotherms for phenanthrene in soils. (**c**)40 °C adsorption isotherms for phenanthrene in soils. (**d**) 20 °C desorption isotherms for phenanthrene in soils. (**e**) 30 °C desorption isotherms for phenanthrene in soils. (**f**) 40 °C desorption isotherms for phenanthrene in soils.

The isotherm was used for quantitative analysis of phenanthrene transport from liquid to solid phase and for understanding the nature of interactions between phenanthrene and the soil matrix. The sorption and desorption isotherms of phenanthrene in soils were shown in Fig. 3. The data showed that phenanthrene adsorption and desorption capacities of three soils varied markedly due to their different physicochemical properties. With the increase of phenanthrene concentration, the adsorbed amount increased. At the same temperature, the adsorption capacity of silty sand was minimum while loess was maximum. This is mainly related to the soil physicochemical properties. At the same initial concentration, the temperature increase from 20 °C to 40 °C showed that the adsorption and desorption capacity decreased with temperature increase. On the one hand, the rise of temperature can increase the phenanthrene solubility in the liquid phase. On the other hand, it could reduce various forces between the soil surface and phenanthrene^37^.

The Freundlich isotherm was used mainly for adsorption surfaces with nonuniform energy distribution, and the Langmuir isotherm was used for monolayer adsorption on perfectly smooth and homogeneous surfaces^38^. The experimental data were fitted with the Langmuir and Freundlich adsorption models, and the isotherm parameters logK_F_, 1/n, K_L_, q_max_ and the coefficient of determination (R^2^) of phenanthrene in soils were listed in Table 4.

**Table 4:** Isotherm parameters for Phenanthrene sorption in soils.

Sample	Study	Temperature (K)	Freundlich model			Langmuir model		
			1/n	logK_F_	R^2^	K_L_	*q*_*max*_ (mg/kg)	R^2^
Loess	Adsorption	293	0.7634	0.0047	0.9649	0.6833	0.8078	0.873
		303	0.1049	0.6175	0.9016	0.8692	1.7677	0.9511
		313	1.1752	0.0246	0.9262	0.8734	0.8203	0.9356
	Desorption	293	6.9771	3.6937	0.9124	0.0101	0.0546	0.859
		303	2.5247	0.9422	0.9074	0.1128	0.2316	0.9352
		313	2.3701	0.8138	0.9056	1.9447	0.2554	0.8498
Silts	Adsorption	293	0.5945	0.0327	0.945	0.2143	0.3826	0.7925
		303	0.7452	0.1205	0.9064	2.8815	2.5893	0.9016
		313	1.0871	0.1572	0.9767	1.2027	2.1445	0.9839
	Desorption	293	1.5482	0.5129	0.9007	1.5262	0.6050	0.8702
		303	3.5035	1.4883	0.9706	2.6918	0.1073	0,9362
		313	1.8718	0.6709	0.9517	0.3343	0.4364	0.9349
Silty sand	Adsorption	293	0.8539	0.0037	0.9251	2.9685	1.2747	0.9647
		303	0.3883	0.2061	0.9501	1.9260	0.8137	0.9549
		313	0.5623	0.3597	0.9092	0.7591	0.7375	0.916
	Desorption	293	1.2672	0.168	0.9107	0.73551	0.9541	0.9097
		303	1.1348	0.0005	0.9124	1.0833	1.5365	0.8503
		313	0.9352	0.1741	0.93	3.7405	4.9652	0.938

As shown in Table 4, according to the coefficients of determination (R^2^), all soils were better fitted with the Freundlich model, which assumes that phenanthrene sorption and desorption occurs on a heterogeneous surface with the possibility of sorption being multi-layered^39^. This phenomenon has also been observed in humic acid and nanometer clay mineral^40^. It showed that the soil adsorption of organic matter was not only surface adsorption but also the process of soil organic matter distribution^41–43^ reached the equilibrium isotherm fitted well with the Freundlich equation when studying the adsorption behavior of aromatic compounds by solids.

#### Adsorption and desorption thermodynamics

**Figure 4:**
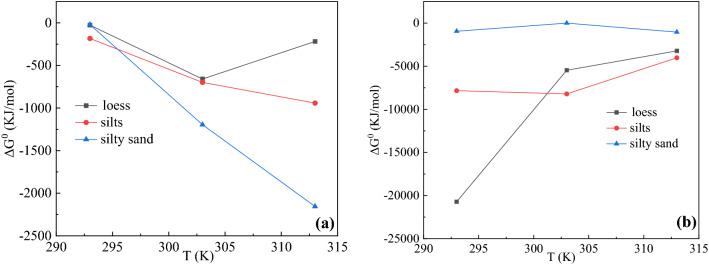
(**a**) Effect of temperature on phenanthrene adsorption free energy in the three tested soils. (**b**) Effect of temperature on phenanthrene desorption free energy in the three tested soils.

To clarify the adsorption mechanisms, the thermodynamic parameters mentioned earlier were calculated and presented in Table 5. Generally, the value of Gibbs free energy changeΔG^0^ indicated the spontaneity of a chemical reaction. Therefore, it could evaluate whether sorption was relate to spontaneous interaction^44^. Negative values of ΔG^0^ indicated that the feasibility and spontaneous nature. The research was under the temperature range about 293–313 K. For adsorption process, all soils ΔG^0^ was < 0 (Fig. 4). It indicated that the processes of adsorption were spontaneous reactions. All adsorption processes were ΔH > 0 and desorption ΔH < 0. It had been reported that adsorption of organic pollutants in a solid–liquid interface was generally caused by a combination of various adsorption forces^45^.

**Table 5:** Thermodynamic parameters for Phenanthrene adsorption in the three tested soils.

sample	Study	K_F_			Δ*G*^0^ (KJ/mol)			Δ*H*^0^(KJ/mol)	Δ*S*^0^(KJ/mol/K)
		293 K	303 K	313 K	293 K	303 K	313 K		
Loess	Adsorption	1.010881	1.298973	1.087176	− 26.3628	− 658.941	− 217.509	0.002494	0.027436
	Desorption	4939.693	8.753868	3.447466	− 20,718.3	− 5465.27	− 3220.69	− 0.00025	0.026605
Silts	Adsorption	1.078202	1.319775	1.43615	− 183.418	− 698.965	− 941.939	0.00582	0.029099
	Desorption	24.93446	26.06754	4.687054	− 7834.79	− 8214.14	− 4020.02	− 0.00075	0.025773
Silty sand	Adsorption	1.008556	1.607311	2.289286	− 20.7537	− 1195.49	− 2155.31	0.002494	0.028268
	Desorption	1.472313	1.001152	1.493138	− 942.329	− 2.90027	− 1043.2	4.99E-05	0.027436

### Response surface analysis

#### Model building and significance testing

In this study, the adsorption rate response value was selected to present the Box-Behnken experimental design and experimental results. The specific results are shown in Table 6.

**Table 6:** Design and results of box Behnken experiment for three soils.

serial number	Each coding level				Loess adsorption rate (%)	Slits adsorption rate (%)	Slity sand adsorption rate (%)
	A	B	C	D			
1	30	6	40	0	81.61	82.02	79.65
2	20	6	20	1	83.05	89.43	81.09
3	30	6	30	− 1	71.6	76.03	78.68
4	20	6	30	0	81.99	83.45	77.11
5	20	6	30	0	80.06	81.36	78.18
6	10	10	30	0	88.41	84.06	84.48
7	30	6	30	1	89.63	93.05	85.66
8	20	6	30	0	81.99	84.06	78.18
9	20	10	30	− 1	73.06	78.08	81.1
10	20	10	20	0	89.01	88.99	84.04
11	20	10	40	0	82.03	82.61	80.1
12	20	6	40	1	89.03	87.01	85.15
13	10	2	30	0	84.45	85.04	79.48
14	10	6	30	1	88.39	90.06	82.44
15	20	2	20	0	88.08	86.63	84.09
16	20	6	20	− 1	75.05	78.02	80.08
17	20	2	30	1	91.06	79.57	89.08
18	20	2	40	0	82.02	84.39	83.12
19	20	10	30	1	92.04	93.02	86.09
20	20	2	30	− 1	79.02	79.57	79.06
21	20	6	30	0	82.02	83.57	77.11
22	30	6	20	0	85.57	84.61	86.62
23	30	2	30	0	86.61	85.05	80.64
24	10	6	40	0	83.36	79.4	76.42
25	20	6	40	− 1	78.02	75.6	75.04
26	10	6	20	0	86.4	85.02	87.43
27	30	10	30	0	89.57	85.41	85.58
28	10	6	30	− 1	86.43	79.1	75.48
29	20	6	30	0	82.02	83.57	77.11

Experiments were carried out according to the design table, and three kinds of experimental data were obtained respectively. Design-expert software is used to optimize the adsorption rate experiment and treatment. According to the results of the experimental design, constant terms, linear terms (A, B, C, D), interaction terms (AB, AC, AD, BC, BD, CD) and square terms (A^2^, B^2^, C^2^, D^2^) on the adsorption rate. The corresponding equations for the following second-order polynomials can be derived from the experimental data obtained:

$$\begin{aligned} {\text{Loess adsorption rate}} & = {8}0.{84} - {1}.0{\text{7A}} + 0.{\text{24B}} - 0.{\text{92C}} + {5}.{\text{84D}} - 0.{\text{25AB}} - 0.{\text{23AC}} + {4}.0{\text{2AD}} \\ &\quad - 0.{\text{23BC}} + {1}.{\text{73BD}} + 0.{\text{75CD}} + 0.{\text{32A}}^{{2}} + {3}.{\text{44B}}^{{2}} + 0.{\text{67C}}^{{2}} - 0.{\text{19D}}^{{2}} , \end{aligned}$$

$$\begin{aligned} {\text{silts adsorption rate}} & = {83}.0{9} + 0.{\text{29A}} - 0.{\text{13B}} - {1}.{\text{81C}} + {6}.{\text{6D}} + 0.{\text{34AB}} + 0.{\text{76AC}} + {1}.{\text{51AD}} \\ &\quad- {1}.0{\text{4BC}} + \, 0.{\text{36BD}} + {\text{CD}} + 0.{\text{17A}}^{{2}} + {2}.{\text{31B}}^{{2}} - 0.{\text{47C}}^{{2}} + 0.{\text{57D}}^{{2}} , \end{aligned}$$

$${\text{silty sand adsorption rate}} = {77}.{9}0 + 0.{\text{93A}} + 0.{\text{49B}} - {1}.{\text{99C}} + {3}.{\text{34D}} - 0.0{\text{1AB}} + {1}.0{\text{1AC}} + {\text{5E}} - 00{\text{3AD}} - 0.{\text{74BC}} + {1}.{\text{26BD}} + {2}.{\text{27CD}} + {1}.{\text{76A}}^{{2}} + {3}.{\text{55B}}^{{2}} + {1}.{\text{79C}}^{{2}} + {1}.{\text{31D}}^{{2}} ,$$

In the formula: A is the concentration of phenanthrene, mg/L; B is pH; C is temperature, °C; D is organic matter, g/kg. A second-order polynomial indicates that the effects of the 4 experimental factors on the response values are interactive, rather than a simple linear relationship (Supplementary Information).

The response surface model is evaluated by variance analysis and significance test to test whether the model can be used to optimize the experimental conditions. The statistical significance of the model equation is determined by the F value, and the significance of each regression coefficient is determined by the P value. The analysis of variance of the model and the fitting results of the quadratic regression equation are shown in Tables 7, 8, 9.

**Table 7:** Variance analysis results of loess optimization experiment.

Source of variance	Sum of Squares	df	Mean Square	F value	P value	Salience
Model	637.56	14	45.54	4.27	0.0052	Significant
A–A phenanthrene concentration	13.76	1	13.76	1.29	0.2751	Not significant
B–B pH	0.69	1	0.69	0.065	0.8028	Not significant
C–C temperature	10.25	1	10.25	0.96	0.3436	Not significant
D–D organic matter	408.57	1	408.57	38.30	< 0.0001	Significant
AB	0.25	1	0.25	0.023	0.8805	Not significant
AC	0.21	1	0.21	0.020	0.8900	Not significant
AD	64.56	1	64.56	6.05	0.0275	Significant
BC	0.21	1	0.21	0.020	0.8900	Not significant
BD	12.04	1	12.04	1.13	0.3060	Not significant
CD	2.27	1	2.27	0.21	0.6520	Not significant
A^2^	59.13	1	59.13	5.54	0.0337	Significant
B^2^	76.56	1	76.56	7.18	0.0180	Significant
C^2^	2.91	1	2.91	0.27	0.6099	Not significant
D^2^	0.23	1	0.23	0.021	0.8861	Not significant
Residual	149.33	14	10.67			
Lack of fit	142.93	10	14.29	8.94	0.0245	Significant
Pure error	6.39	4	1.60			
Cor total	786.88	28

**Table 8:** Analysis of variance results of silts optimization experiment.

Source of variance	Sum of squares	df	Mean square	F value	P value	Salience
Model	620.72	14	44.34	30.37	< 0.0001	Significant
A-A phenanthrene concentration	1.02	1	1.02	0.70	0.4184	Not significant
B-B pH	0.20	1	0.20	0.14	0.7149	Not significant
C–C Temperature	39.13	1	39.13	26.81	0.0001	Significant
D-D Organic matter	522.98	1	522.98	358.27	< 0.0001	Significant
AB	0.45	1	0.45	0.31	0.5880	Not significant
AC	2.30	1	2.30	1.57	0.2304	Not significant
AD	9.18	1	9.18	6.29	0.0251	Significant
BC	4.28	1	4.28	2.94	0.1087	Not significant
BD	0.53	1	0.53	0.37	0.5554	Not significant
CD	0.000	1	0.000	0.000	1.0000	Not significant
A^2^	0.20	1	0.20	0.13	0.7195	Not significant
B^2^	34.47	1	34.47	23.61	0.0003	Significant
C^2^	1.41	1	1.41	0.97	0.3424	Not significant
D^2^	2.11	1	2.11	1.44	0.2494	Not significant
Residual	20.44	14	1.46			
Lack of Fit	16.14	10	1.61	1.50	0.3699	Not significant
Pure Error	4.30	4	1.07			
Cor Total	641.16	28

**Table 9:** Analysis of variance results of silty sand optimization experiment.

Source of variance	Sum of squares	df	Mean square	F value	P value	Salience
Model	324.37	14	23.17	3.88	0.0081	Significant
A-A phenanthrene concentration	10.27	1	10.27	1.72	0.2110	Not significant
B-B pH	2.92	1	2.92	0.49	0.4959	Not significant
C–C temperature	47.48	1	47.48	7.95	0.0137	Significant
D-D organic matter	133.80	1	133.80	22.39	0.0003	Significant
AB	9.000E–004	1	9.000E–004	1.506E–004	0.9904	Not significant
AC	4.08	1	4.08	0.68	0.4224	Not significant
AD	1.000E–004	1	1.000E–004	1.674E–005	0.9968	Not significant
BC	2.21	1	2.21	0.37	0.5532	Not significant
BD	6.33	1	6.33	1.06	0.3210	Not significant
CD	20.70	1	20.70	3.46	0.0838	Not significant
A^2^	20.08	1	20.08	3.36	0.0881	Not significant
B^2^	81.62	1	81.62	13.66	0.0024	Significant
C^2^	20.86	1	20.86	3.49	0.0827	Not significant
D^2^	11.10	1	11.10	1.86	0.1943	Not significant
Residual	83.65	14	5.97			
Lack of fit	82.85	10	8.28	41.27	0.0013	Significant
Pure error	0.80	4	0.20			
Cor total	408.02	28				

According to Tables 7, 8, 9, the adaptability of the three soil models was very significant (F > 1, P < 0.05). Among them, the organic matter P value of loess is less than 0.0001, indicating that it has a very significant effect on the adsorption rate of loess, and the P value of AD is less than 0.05, which indicates that the interaction between phenanthrene concentration and organic matter has a more significant effect on the adsorption rate, and its complex correlation coefficient of determination R^2^ = 0.8102, indicating that the fit of the response model is good, and the experimental error is within the acceptable range. The fitting degree of the silt prediction model is R^2^ = 0.9681, indicating that the model fits well with the experimental results and the experimental accuracy is high; the correction coefficient of determination R^2^Adj = 0.9363, indicating that about 93.63% of the response value changes can be explained by this model; at the same time, by From the F value, it can be obtained that the order of the influence of various factors on the silt adsorption rate is organic matter > temperature > phenanthrene concentration > pH. In the interaction, the phenanthrene concentration and organic matter have a significant effect on the silt adsorption rate. The coefficient of determination of the silt complex correlation is R^2^ = 0.9464, indicating that the response model has a good fit, and the experimental error is within the acceptable range. Adjusting the complex correlation coefficient R^2^ = 0.8982 indicates that the regression relationship can explain 89.82% of the change in the dependent variable. Therefore, this The model can be used to analyze and predict the effect of different factors on the adsorption rate of phenanthrene.

#### 3D response surface analysis

In response surface optimization, the three-dimensional response surface graph reflects the influence of the interaction of the other two variables on the response value, and the slope of the response surface reflects the significance of the interaction of the two variables on the response value. The more significant the interaction effect is on the response value, when the slope is gentle, the effect is not significant. If the contour map is elliptical, it indicates that the interaction between the two variables is significant, and if the contour map is circular, it is not significant^46^. In addition, the slope and density of the contour line also reflect the influence of the variable on the response value. The steeper the contour line and the greater the density, the greater the influence of the variable on the response value^47^.

**Figure 5:**
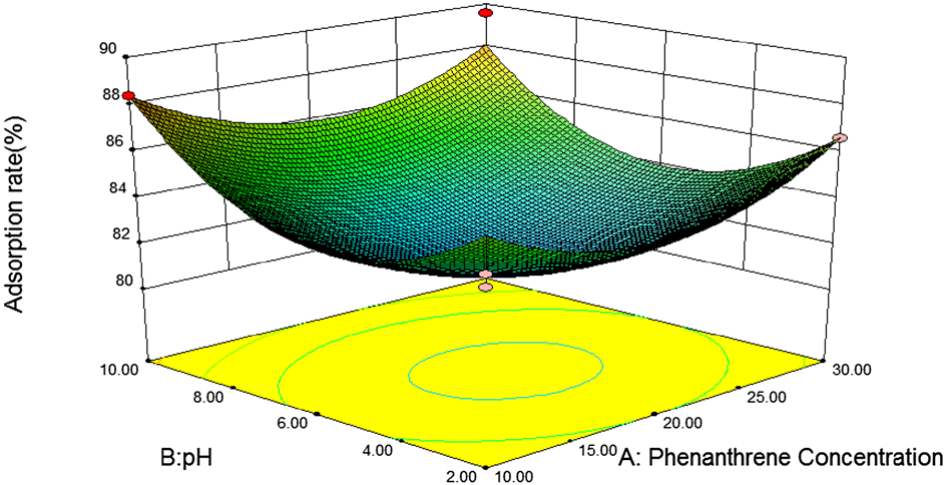
Effects of initial phenanthrene concentration and pH on adsorption rate of loess.

**Figure 6:**
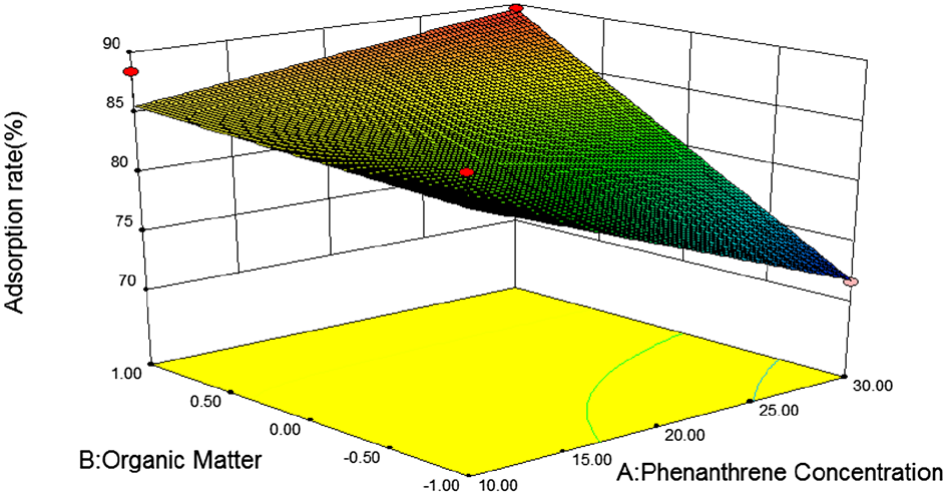
Effects of initial phenanthrene concentration and organic matter on the adsorption rate of loess.

**Figure 7:**
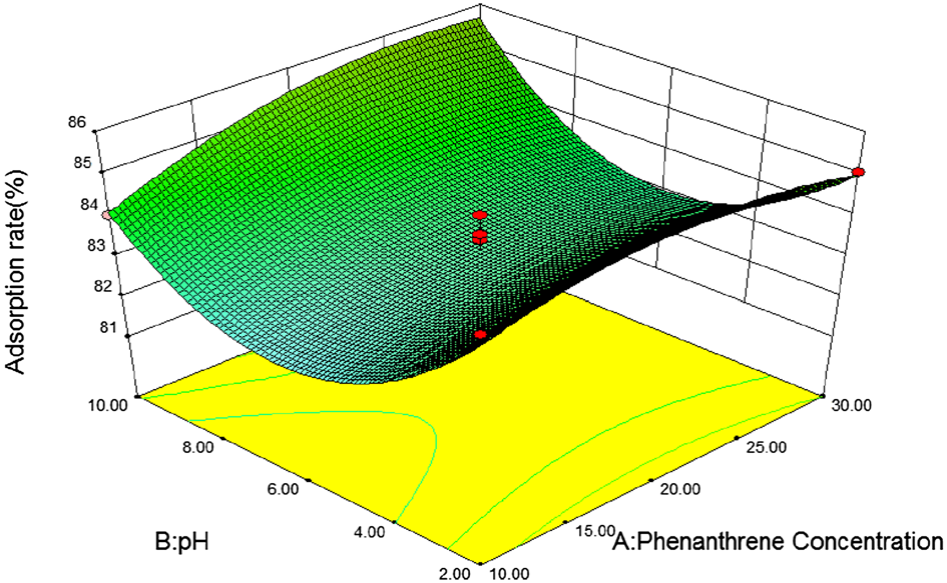
Effects of initial phenanthrene concentration and pH on silts adsorption rate.

**Figure 8:**
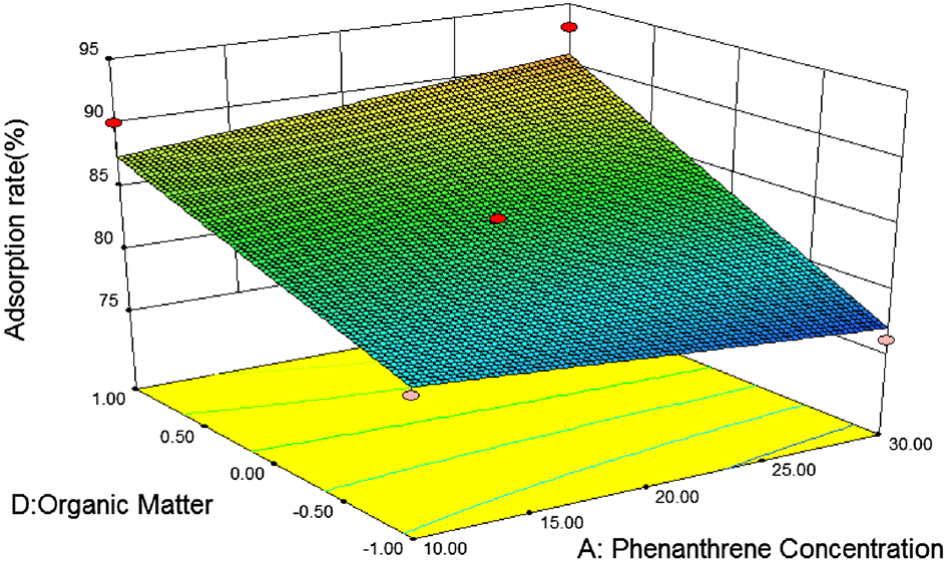
Effects of soil organic matter and phenanthrene concentration on silts adsorption rate.

**Figure 9:**
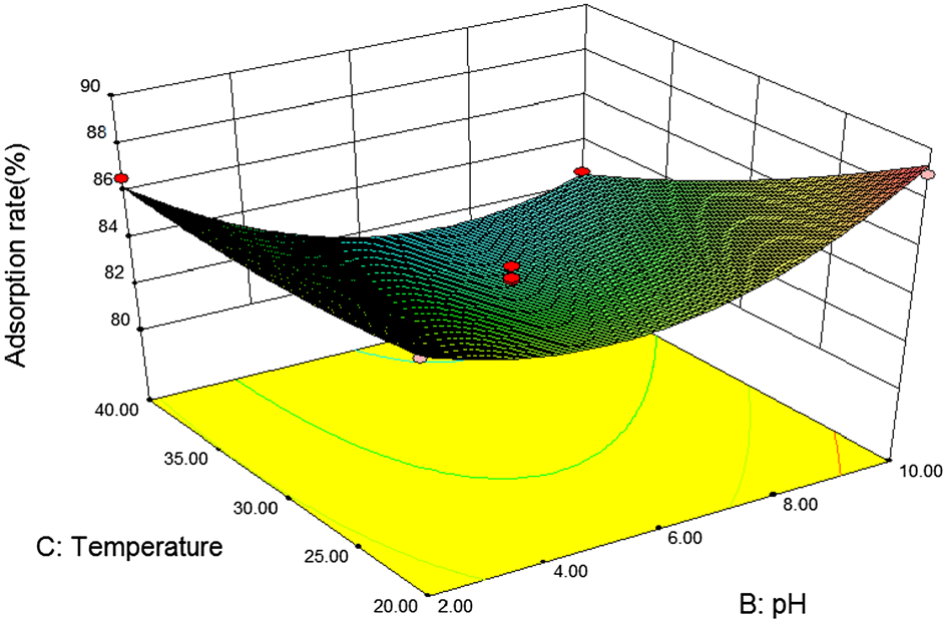
Effects of temperature and pH on the adsorption rate of silts.

**Figure 10:**
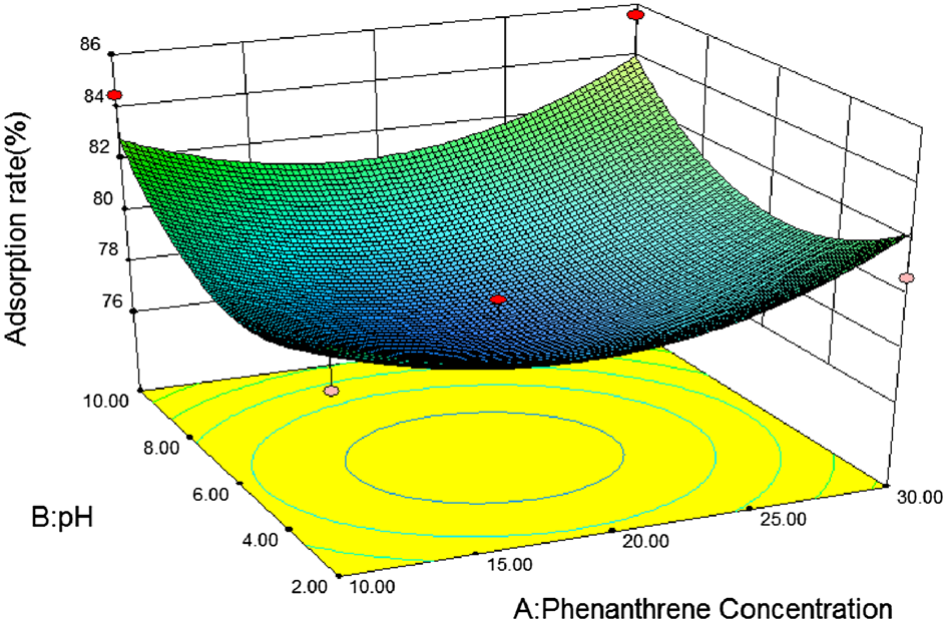
Effects of initial phenanthrene concentration and pH on silty sand adsorption rate.

- (1) *Loess* Fig. 5 is a three-dimensional response surface diagram of the interaction between initial phenanthrene concentration and pH to phenanthrene adsorption on loess. It can be seen from the figure that the slope of the response surface graph is steep, and the contour line is an approximate circle, indicating that the interaction between phenanthrene concentration and pH is not significant for the response value. With the increase of pH, the adsorption rate of phenanthrene on loess showed a slow decline at first to the lowest point at 6, and then gradually increased. When the soil pH was close to 6, with the increase of the initial phenanthrene concentration, the adsorption rate of loess also showed a trend of first decreasing and then increasing. According to the F value, F = 0.337, P = 0.5532 > 0.05, it can be concluded that soil pH and initial phenanthrene concentration of the solution have no significant interaction on the adsorption rate of loess.
- Figure 6 shows the effects of initial phenanthrene concentration and organic matter on phenanthrene adsorption on Loess under the condition that pH value and temperature are at the central point. It can be seen from the figure that the initial phenanthrene concentration and soil organic matter contour are steep, indicating that their interaction is significant. The range of phenanthrene adsorption rate is 70 ~ 95, and the change of surface is steep. From the Loess error analysis, it can be seen that if f value is 6.05 and P value is 0.0275 < 0.05, the interaction between initial phenanthrene concentration and soil organic matter on Loess adsorption rate is more significant. In the 3D response surface diagram, the influence of soil organic matter on the adsorption rate of loess is more significant, and the adsorption rate of loess also increases with the increase of organic matter content, that is, when the coding value of loess soil organic matter is 1 and the concentration of phenanthrene is 30 mg/L, it is the best reaction condition of loess.
- It can be seen from Table 8 that after optimization by the response surface method, the best influencing conditions for the adsorption of phenanthrene in loess are pH = 10, initial phenanthrene concentration of 30 mg/L, temperature of 20.14 °C, soil organic matter content code value of 1, theoretical energy The best adsorption rate achieved was 98.89%^48^.
- (2) *Slits* Fig. 7 is a three-dimensional response surface diagram of initial phenanthrene concentration and pH to phenanthrene adsorption by silt under central conditions of temperature and organic matter.It can be seen from Fig. 3 that the adsorption rate of silts to phenanthrene decreases first and then increases with the increase of pH value, indicating that the increase of pH is conducive to the collision between phenanthrene molecules and soil, and then the adsorption of phenanthrene to the soil increases. Adsorption rate; with the increase of phenanthrene concentration, the adsorption rate changed little, which may be because the concentration of phenanthrene concentration in the experimental area was higher, which exceeded the adsorption saturation of silts to phenanthrene, so the adsorption rate remained basically unchanged.
- Figure 8 effects of initial phenanthrene concentration and organic matter on silts adsorption rate. It can be seen from Fig. 8 that the adsorption rate of silts to phenanthrene increases with the increase of organic matter content, because the increase of organic matter content can provide more adsorption sites to adsorb phenanthrene, thus making the adsorption rate increase; With the increase of concentration, the change of adsorption rate is small, which may be because the concentration of phenanthrene concentration in the experimental area is higher, which exceeds the adsorption saturation of silts to phenanthrene, so that the adsorption rate remains basically unchanged. According to the F value, F = 6.29, P = 0.0251 < 0.05, that is to say, it can be concluded that the initial phenanthrene concentration and organic matter interact significantly on the silts adsorption rate.
- Figure 9 shows the effects of temperature and pH on the adsorption rate of silts under the initial phenanthrene concentration and soil organic matter in the central condition. It can be seen from Fig. 9 that the trend of the contour map of temperature and pH is relatively slow, which means that the interaction effect of temperature and pH on the adsorption rate of silts is low; the adsorption rate of silts to phenanthrene decreases with the increase of temperature. With the increase of pH, the adsorption of silts first decreased and then increased. When pH = 6, the adsorption rate of silts was the lowest. It can be seen from Table 8 that the F value of temperature and pH is 2.94, and the P value is 0.1087, indicating that the interaction between temperature and pH is not significant.
- It can be seen from Table 10 that after the optimization of the response surface method, the optimal conditions for the adsorption of phenanthrene by the silts are pH = 10, the initial phenanthrene concentration is 30 mg/L, the temperature is 21.15 °C, the soil organic matter content code value is 1, and the theoretical value is 1. The best adsorption rate that can be achieved is 96.59%.
- (3) *Silty sand* When the temperature is 30 °C and the organic matter is 0, the effect of initial phenanthrene concentration and solution pH on the adsorption rate of silty sand is shown in Fig. 10. The trend of silty sand adsorption rate and loess adsorption rate is roughly similar. With the increase of pH value, when the pH value of silty sand is close to 6, the adsorption rate of silty sand is the lowest. With the increase of initial phenanthrene concentration, the adsorption rate of silty sand alsodecreased first and then increased. When pH = 6 and phenanthrene concentration was 20 mg/L,the adsorption rate of silty sand to phenanthrene was the lowest.

**Table 10:** Verification results of adsorption rate test.

Test conditions	The actual adsorption rate of loess %	Predicted adsorption rate of loess %	Relative error	The actual adsorption rate of silts %	Predicted adsorption rate of silts %	Relative error	The actual adsorption rate of silty sand %	Predicted adsorption rate of silty sand %	Relative error
(1)	95.45	98.89	3.44	94.33	96.59	2.26	89.44	93.37	3.93
(1)	94.37	98.89	4.52	93.5	96.59	3.09	92.14	93.37	1.23
(1)	95.68	98.89	3.21	92.98	96.59	3.61	90.87	93.37	2.5
(2)	93.27	94.96	1.63	89.27	93.98	4.71	80.18	83.66	3.48
(2)	90.69	94.96	4.27	93.5	93.98	0.48	80.7	83.66	2.96
(2)	89.99	94.96	4.97	93.53	93.98	0.45	81.79	83.66	1.87
(3)	90.44	89.46	1.98	87.86	91.99	4.13	73.66	76.71	3.05
(3)	87.92	89.46	1.54	89.65	91.99	2.34	72.43	76.71	4.28
(3)	86.63	89.46	2.83	88.35	91.99	3.64	74.18	76.71	2.53

**Figure 11:**
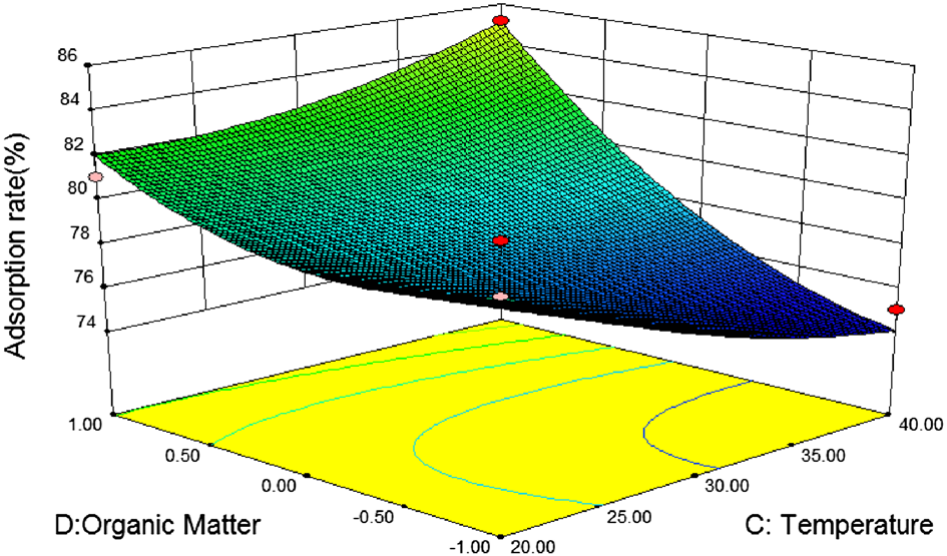
Effects of soil organic matter and temperature on silty sand adsorption rate.

Figure 11 shows the effects of temperature and soil organic matter on the adsorption rate of phenanthrene under the condition of pH value and initial phenanthrene concentration at the center point. It can be seen from the figure that the temperature and soil organic matter contour is steep, indicating that the interaction is more significant. The range of adsorption rate of phenanthrene by silty sand is 70 ~ 85, and the surface change is steep. It can be seen from the analysis of silty sand variance that the F value is 3.46 and the P value is 0.0838. It can be seen from Fig. 11 that soil organic matter has a significant effect on the adsorption rate of silty sand, and the adsorption rate of silty sand increases with the increase of organic matter content. That is, when the organic matter coding value of silty sand soil is 1 and the temperature is 20 °C, it is the best reaction condition for silty sand.

**Figure 12:**
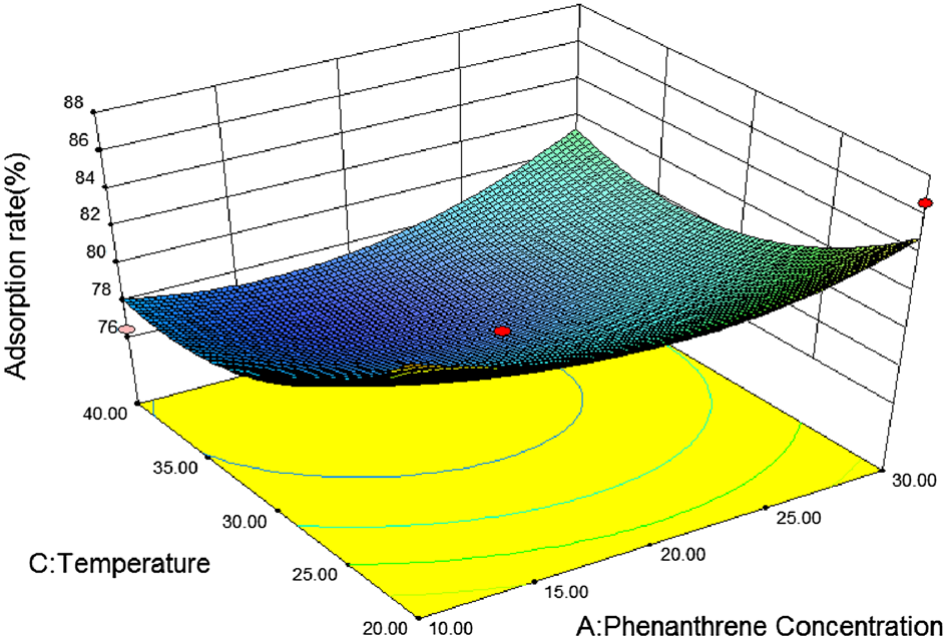
Effects of initial phenanthrene concentration and temperature on silty sand adsorption rate.

Figure 12 shows the effects of initial phenanthrene concentration and temperature on the adsorption rate of phenanthrene under the condition of pH value and organic matter at the central point. It can be seen from the figure that the interaction between initial phenanthrene concentration and temperature is not significant, the range of phenanthrene adsorption rate is 76 ~ 84, and the surface change is gentle. When the temperature is 20 °C, the initial concentration of phenanthrene is 30 mg/L, and the adsorption rate of phenanthrene decreases with the increase of temperature.

It can be seen from Table 10 that after optimization by the response surface method, the optimum conditions for the adsorption of phenanthrene in silty sand are pH = 2, the initial phenanthrene concentration is 29.96 mg/L, the temperature is 40 °C, and the code value of soil organic matter content is 1. The optimal adsorption rate that can be achieved theoretically is 93.37%.

#### Model validation

In order to verify the reliability of the response model, three groups of experiments were designed for verification. Loess experimental conditions (1) Using the quadratic regression linear equation obtained by the Design Expert8.0 program, the optimal experimental conditions were obtained by simulation: pH = 10, initial phenanthrene concentration of 30 mg/L, temperature of 20.14 °C, soil organic matter content code The value is 1; (2) pH = 2, the initial phenanthrene concentration is 30 mg/L, the temperature is 20 °C, and the soil organic matter content code value is 1; (3) pH = 2, the initial phenanthrene concentration is 10 mg/L, and the temperature is At 20 °C, the coded value of soil organic matter content is − 1. Silts experimental conditions (1) Using the quadratic regression linear equation obtained by the Design Expert8.0 program, the optimal experimental conditions were obtained by simulation: pH = 10, the initial phenanthrene concentration was 30 mg/L, the temperature was 21.15 °C, and the soil organic matter content was The code value is 1; (2) pH = 2, the initial phenanthrene concentration is 30 mg/L, the temperature is 30.12 °C, and the code value of soil organic matter content is 1; (3) pH = 2, the initial phenanthrene concentration is 23.9 mg/L, The temperature is 40 °C, and the coded value of soil organic matter content is 1. Silty sand experimental conditions (1) Using the quadratic regression linear equation obtained by the Design Expert8.0 program, the optimal experimental conditions were obtained by simulation: pH = 2, the initial phenanthrene concentration was 29.96 mg/L, the temperature was 40 °C, and the soil organic matter The content code value is 1; (2) pH = 2, the initial phenanthrene concentration is 29.96 mg/L, the temperature is 40 °C, and the soil organic matter content code value is 0; (3) pH = 2, the initial phenanthrene concentration is 29.96 mg/L, the temperature is 40 °C, the soil organic matter content code value is − 1. In order to investigate the practicability and accuracy of the optimization results, three parallel tests were carried out to verify the adsorption of phenanthrene by soil under the best conditions.

The actual measured average values are as follows. The test results are shown in Table 10.

The model verification results are shown in Table 10. The relative errors between the experimental results and the predicted values of the adsorption rate of phenanthrene in loess, silts and silty sand are all less than 5%, which indicates that the model is suitable and effective. The adsorption process of PAHs has certain guiding significance.

## Conclusion

Phenanthrene adsorption and desorption on three soils in Guanzhong Basin were studied using a batch technique. The adsorption and desorption kinetics data were better modeled using pseudo-second-order kinetic equations, indicating that the rate-limiting step was chemical adsorption and the sorption was governed by availability of sorption sites on soil surfaces. Phenanthrene adsorption and desorption isotherms in soils were well correlated with Freundlich models. Moreover, the calculated ΔH and ΔG values indicated that the adsorption process was endothermic reaction and desorption process was exothermic reaction. The influencing factors of adsorption experiment were optimized by response surface method. The results showed that the best influencing conditions for the adsorption of phenanthrene on loess were pH = 10, the initial phenanthrene concentration was 30 mg/L, the temperature was 20.14 °C, the coding value of soil organic matter content was 1, and the best adsorption rate that could be achieved in theory was 98.89%. The optimum conditions for phenanthrene adsorption by silts are pH = 10, initial phenanthrene concentration of 30 mg/L, temperature of 21.15 °C, code value of soil organic matter content of 1, and the best adsorption rate that can be achieved in theory is 96.59%. The best influencing conditions for phenanthrene adsorption by silty sand are pH = 2, the initial phenanthrene concentration is 29.96 mg/L, the temperature is 40 °C, the coding value of soil organic matter content is 1, and the best adsorption rate that can be achieved in theory is 93.37%.

## Date availability

The datasets used and/or analysed during the current study available from the corresponding author on reasonable request.

## Supplementary Information

Supplementary Information.
