# Supplementary material for: Analysis of influencing factors of phenanthrene adsorption by different soils in Guanzhong basin based on response surface method
Source: Sci Rep. 2022 Dec 3;12:20906. doi: 10.1038/s41598-022-25293-0 (PMC9719479; doi:10.1038/s41598-022-25293-0)
Supplement: Supplementary file 1 — Supplementary Information. [file 41598_2022_25293_MOESM1_ESM.pdf]

Analysis of influencing factors of phenanthrene adsorption by different soils in Guanzhong Basin Based on response surface method

Hua Tian[*a,b], Qing Zhang[a], Xue Tian[c], Zu-feng Xie [a], Fang Pu [a] ,Qian-ji Wang [a]

[a] College of Geology and Environment, Xi'an University of Science and Technology, Xi'an, 710054, China

[b] Shaanxi Provincial Key Laboratory of Green Coal Development and Geological Guarantee, Xi'an, 710054, China

[c] Zhongsheng Environmental Technology Development Co,Ltd, Xi'an, 710054, China

(* Corresponding author: +86 180 6663 5829; E-mail: tianhua@xust.edu.cn)

Original data

1.Original data of the influence of initial phenanthrene concentration and pH value on the adsorption rate of loess.

Select	Std	Run	Factor 1 A:A phenant... mg·L-1	Factor 2 B:B pH	Factor 3 C:C tempera... ℃	Response 1 Adsorption r... %
	16	1	20.00	6.00	30.00	82.02
	13	2	20.00	6.00	30.00	81.99
	14	3	20.00	6.00	30.00	80.06
	7	4	10.00	6.00	40.00	83.36
	6	5	30.00	6.00	20.00	85.57
	2	6	30.00	2.00	30.00	86.61
	15	7	20.00	6.00	30.00	81.99
	5	8	10.00	6.00	20.00	86.4
	4	9	30.00	10.00	30.00	89.57
	10	10	20.00	10.00	20.00	89.01
	8	11	30.00	6.00	40.00	81.61
	3	12	10.00	10.00	30.00	88.41
	1	13	10.00	2.00	30.00	84.45
	11	14	20.00	2.00	40.00	82.02
	12	15	20.00	10.00	40.00	82.03
	9	16	20.00	2.00	20.00	88.08
	17	17	20.00	6.00	30.00	82.02

2.Original data of the influence of initial phenanthrene concentration and pH value on the adsorption rate of Silty sand.

Select	Std	Run	Factor 1 A:A Phenant... mg·L-1	Factor 2 B:B pH	Factor 3 C:C Temper... ℃	Response 1 Adsorption r... %
	8	1	30.00	6.00	40.00	79.65
	3	2	10.00	10.00	30.00	84.48
	15	3	20.00	6.00	30.00	78.18
	17	4	20.00	6.00	30.00	77.11
	2	5	30.00	2.00	30.00	80.64
	14	6	20.00	6.00	30.00	78.18
	13	7	20.00	6.00	30.00	77.11
	7	8	10.00	6.00	40.00	76.42
	1	9	10.00	2.00	30.00	79.48
	16	10	20.00	6.00	30.00	77.11
	9	11	20.00	2.00	20.00	84.09
	11	12	20.00	2.00	40.00	83.12
	5	13	10.00	6.00	20.00	87.43
	4	14	30.00	10.00	30.00	85.58
	12	15	20.00	10.00	40.00	80.1
	6	16	30.00	6.00	20.00	86.62
	10	17	20.00	10.00	20.00	84.04

3.Original data of the influence of initial phenanthrene concentration and pH value on the adsorption rate of Slits.

Select	Std	Run	Factor 1 A:A Phenant... mg·L-1	Factor 2 B:B pH	Factor 3 C:C Temper... ℃	Response 1 Adsorption r... %
	14	1	20.00	6.00	30.00	81.36
	16	2	20.00	6.00	30.00	83.57
	1	3	10.00	2.00	30.00	85.04
	2	4	30.00	2.00	30.00	85.05
	6	5	30.00	6.00	20.00	84.61
	5	6	10.00	6.00	20.00	85.02
	10	7	20.00	10.00	20.00	88.99
	12	8	20.00	10.00	40.00	82.61
	9	9	20.00	2.00	20.00	86.63
	17	10	20.00	6.00	30.00	83.57
	13	11	20.00	6.00	30.00	83.45
	8	12	30.00	6.00	40.00	82.02
	15	13	20.00	6.00	30.00	84.06
	11	14	20.00	2.00	40.00	84.36
	4	15	30.00	10.00	30.00	85.41
	3	16	10.00	10.00	30.00	84.06
	7	17	10.00	6.00	40.00	79.4
